# Novel Chaperones *Rr*GroEL and *Rr*GroES for Activity and Stability Enhancement of Nitrilase in *Escherichia coli* and *Rhodococcus ruber*

**DOI:** 10.3390/molecules25041002

**Published:** 2020-02-24

**Authors:** Chunmeng Xu, Lingjun Tang, Youxiang Liang, Song Jiao, Huimin Yu, Hui Luo

**Affiliations:** 1 Key Laboratory of Industrial Biocatalysis, Ministry of Education, Beijing 100084, China; xu-cm18@mails.tsinghua.edu.cn (C.X.); 13716806298@163.com (L.T.); 13718806596@126.com (Y.L.); jiao14789800892@163.com (S.J.); 2 Department of Chemical Engineering, Tsinghua University, Beijing 100084, China; 3 Center for Synthetic and Systems Biology, Tsinghua University, Beijing 100084, China; 4 Department of Biological Science and Engineering, University of Science and Technology Beijing, Beijing 100083, China; luohui@ustb.edu.cn

**Keywords:** nitrilase, chaperone, stability, *E. coli*, *R. ruber*

## Abstract

For large-scale bioproduction, thermal stability is a crucial property for most industrial enzymes. A new method to improve both the thermal stability and activity of enzymes is of great significance. In this work, the novel chaperones *Rr*GroEL and *Rr*GroES from *Rhodococcus ruber*, a nontypical actinomycete with high organic solvent tolerance, were evaluated and applied for thermal stability and activity enhancement of a model enzyme, nitrilase. Two expression strategies, namely, fusion expression and co-expression, were compared in two different hosts, *E. coli* and *R. ruber*. In the *E. coli* host, fusion expression of nitrilase with either *Rr*GroES or *Rr*GroEL significantly enhanced nitrilase thermal stability (4.8-fold and 10.6-fold, respectively) but at the expense of enzyme activity (32–47% reduction). The co-expression strategy was applied in *R. ruber* via either a plasmid-only or genome-plus-plasmid method. Through integration of the nitrilase gene into the *R. ruber* genome at the site of nitrile hydratase (NHase) gene via CRISPR/Cas9 technology and overexpression of *Rr*GroES or *Rr*GroEL with a plasmid, the engineered strains *R. ruber* TH3 dNHase::*Rr*Nit (pNV18.1-P*ami*-*Rr*Nit-P*ami*-*Rr*GroES) and TH3 dNHase::*Rr*Nit (pNV18.1-P*ami*-*Rr*Nit-P*ami*-*Rr*GroEL) were constructed and showed remarkably enhanced nitrilase activity and thermal stability. In particular, the *Rr*GroEL and nitrilase co-expressing mutant showed the best performance, with nitrilase activity and thermal stability 1.3- and 8.4-fold greater than that of the control TH3 (pNV18.1-P*ami*-*Rr*Nit), respectively. These findings are of great value for production of diverse chemicals using free bacterial cells as biocatalysts.

## 1. Introduction

Nitrilases are enzymes that can convert nitriles to the corresponding acid and ammonia [1,2,3,4]. Nitrilases have attracted the attention of many researchers because of their benefits as catalysts, such as mild reaction conditions, environmental friendliness, high specificity and selectivity compared with traditional chemical approaches [4,5]. Nitrilases are important industrial enzymes due to their wide applications in production of valuable fine or chiral chemicals, such as acrylic acid, which is used to synthesize the valuable polyacrylic acid [4]. In particular, nitrilase from *Rhodococcus rhodochrous* tg1-A6 has been highlighted in previous research with respect to industrial application [6,7].

However, certain problems still need to be resolved for nitrilase application. For example, converting acrylonitrile to acrylic acid is an exothermic reaction, and thus, the operation temperature increases as the reaction proceeds [8]. Therefore, better thermal stability of nitrilase is required to maintain high reaction rates [9]. However, most mesophilic nitrile-degrading enzymes are inactivated rapidly over 50 °C [8]. The low thermal stability of nitrilase has limited its industrial application [8]. Therefore, in previous studies, methods such as point mutations in nitrilase have been reported to improve its activity and/or stability [10,11,12].

Molecular chaperones are special proteins that can aid correct and efficient folding of newly synthesized proteins, minimize aggregation during folding and assist in refolding of denatured proteins [13,14,15]. Many studies have concentrated on applications of the GroES-GroEL chaperone team for protein stabilization, particularly *Ec*GroEL and *Ec*GroES from *E. coli*. For example, with *Ec*GroEL-*Ec*GroES overexpression, the stability of Cryj2 significantly increased compared with the wild-type protein [16]. The activity of nitrile hydratase (NHase) remained at 229 U/L with *Ec*GroEL-*Ec*GroES protection, while that of the control enzyme without chaperones dropped to zero after heat shock at 50 °C for 10 min [17]. Besides, overexpression of *Ec*GroEL-*Ec*GroES in *R. ruber* could stabilize NHase after heat shock [17]. Co-expression of GroEL-GroES was able to increase the solubility and activity of nitrilase from *Penicillium marneffei* [18]. It has also been reported that co-expression of GroEL-GroES significantly increased soluble and functional expression of human interferon-gamma [19]. GroEL (~60 kDa) chaperonins consist of two stacked rings and have a structure similar to that of a cylinder with a central cavity [15,20]. The dome-shaped *Ec*GroES (~10 kDa) serves as a cofactor of *Ec*GroEL, helping to close *Ec*GroEL’s cavity, protect it from proteolytic truncation and trigger its conformational changes [20,21].

*Rhodococcus* spp. are good candidates for nitrile degradation because of their excellent adaptive ability, high organic solvent tolerance and abundant metabolic system, enabling them to degrade and assimilate various organic compounds, such as nitriles [22,23]. In 2018, Chen et al. found that fusion expression of *Rr*GroEL2 (from *R. ruber*) with NHase in *E. coli* significantly improved the enzyme activity and stability [14]. Wang et al. found that a small heat shock protein from *R. ruber* (*Rr*Hsp16) improved the stress tolerance and cell integrity of the host strain [24]. *R. ruber* GroEL analogues were regarded as different from conventional *E. coli* GroEL but more likely to have similar characteristics with two *Mycobacterium tuberculosis* GroEL analogues (*Mt*GroEL1 and *Mt*GroEL2) [14]. *Mt*GroEL1 and *Mt*GroEL2 were reported that their refolding activity was independent of the GroES [25]. Therefore, examining the characteristics and function of *Rr*GroEL and *Rr*GroES for enzymes with promising industrial application, such as nitrilase, is of great interest.

In this work, with nitrilase from *R. rhodochrous* tg1-A6 (*Rr*Nit) as the model enzyme, the novel *Rr*GroEL and *Rr*GroES chaperones were investigated, and their activity and thermal stability enhancement of *Rr*Nit were observed in both *E. coli* and *R. ruber* hosts.

## 2. Results

### 2.1. Fusion Expression of Either RrGroES or RrGroEL with RrNit Can Enhance the Enzyme Thermal Stability in E. coli

Transcriptome analyses of *R. ruber* TH were performed under both urea-induction and heat shock conditions [14,26]. Transcription level changes in the chaperone genes *RrgroES* and *RrgroEL* were highlighted with *RrgroEL2* as a control (Figure S1). Here, urea (10 g/L in the medium) was a special inducer for the high expression of native NHase, amidase, and certain other important enzymes in *R. ruber.* After urea-induction, the transcription level of *RrgroES*, *RrgroEL*, and *RrgroEL2* increased by 25-, 10-, and 32-fold, respectively (Figure S1a); after heat shock, the transcription level of the three chaperone genes was enhanced by 100%, 100%, and 3-fold, respectively (Figure S1b) [14]. These results indicate that *Rr*GroES and *Rr*GroEL, together with *Rr*GroEL2, may play dominant roles in overexpression and thermal stability of intracellular enzymes in *R. ruber* TH. Therefore, they were specifically highlighted in this work.

Amino acid sequence alignment of *Rr*GroES with *Ec*GroES, and *Rr*GroELs with *Ec*GroEL was performed. The amino acid sequence of *Rr*GroES (97 amino acids) shared only ~46% identity with *Ec*GroES (96 amino acids). In addition, for *Rr*GroEL (538 amino acids), the identity rate compared with either *Ec*GroEL (545 amino acids) or *Rr*GroEL2 (541 amino acids) was just ~60%. *Rr*GroEL showed higher identity with *Mt*GroEL1 (approximately 80%) [14]. *Mt*GroEL1 was reported that its refolding activity was independent of the GroES [25]. *Rr*GroES and *Rr*GroEL can be recognized as novel chaperones that likely possess unique positive functions for activity and stability enhancement of the target enzyme. Therefore, the stabilization effect of *Rr*GroES and *Rr*GroEL on intracellular *Rr*Nit was investigated and subsequently compared in both *E. coli* and *R. ruber* hosts.

**Figure 1:**
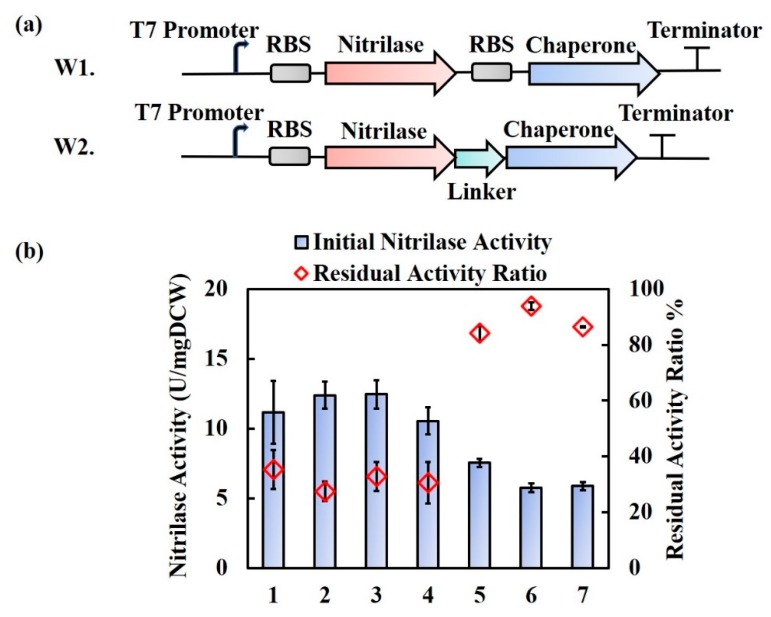
Co-expression and fusion-expression strategies for nitrilase and the chaperones *Rr*GroES, *Rr*GroEL, and *Rr*GroEL2. (**a**) Genetic design for co-expression and fusion expression of nitrilase with chaperones; (**b**) Nitrilase activity and the residual activity ratio of nitrilase in *E. coli* BL21(DE3) recombinant strains. Lane 1 to lane 7: (pET28a-*Rr*Nit) (control), (pET28a-*Rr*Nit+*Rr*GroES), (pET28a-*Rr*Nit+*Rr*GroEL), (pET28a-*Rr*Nit+*Rr*GroEL2), (pET28a-*Rr*Nit-FxL1-*Rr*GroES), (pET28a-*Rr*Nit-FxL1-*Rr*GroEL), and (pET28a-*Rr*Nit-FxL1-*Rr*GroEL2), respectively. The residual activity was measured under 50 °C heat shock for 30 min. Residual activity ratio = Residual nitrilase activity/Initial nitrilase activity.

*E. coli* is one of the most popular hosts in genetic engineering. Separate overexpressions of the three *Rhodococcus* chaperones (*Rr*GroES, *Rr*GroEL, and *Rr*GroEL2) with nitrilase (*Rr*Nit) in *E. coli* were performed in two different ways (Figure 1a). In way 1 (W1), each chaperone was separately co-expressed with nitrilase under the same T7 promotor. In way 2 (W2), each chaperone was fusion-expressed with nitrilase using the linker (GGGGS)_1_ (named FxL1). In W1, three recombinant *E. coli* strains were constructed: BL21(DE3) (pET28a-*Rr*Nit+*Rr*GroES), BL21(DE3) (pET28a-*Rr*Nit+*Rr*GroEL), and BL21(DE3) (pET28a-*Rr*Nit+*Rr*GroEL2); in W2, three recombinant strains were obtained: BL21(DE3) (pET28a-*Rr*Nit-FxL1-*Rr*GroES), BL21(DE3) (pET28a-*Rr*Nit-FxL1-*Rr*GroEL), and BL21(DE3) (pET28a-*Rr*Nit-FxL1-*Rr*GroEL2). *E. coli* BL21(DE3) (pET28a-*Rr*Nit) expressing the nitrilase without a chaperone was used as a control. Intracellular nitrilase activity and the thermal stability of nitrilase were measured, and the results are compared in Figure 1b.

In W1, after co-expression of nitrilase with *Rr*GroES, *Rr*GroEL, and *Rr*GroEL2, individually, no significant changes in nitrilase activity nor residual activity were observed for any of the co-expression strains.

In W2, the activity and thermal stability results were remarkably different (Figure 1b). In short, the individual fusion expression of *Rr*GroES, *Rr*GroEL, and *Rr*GroEL2 with *Rr*Nit significantly enhanced the thermal stability of nitrilase. For example, the residual activity ratio after heat shock increased from 35% to as high as 84% for the *Rr*Nit-FxL1-*Rr*GroES chimera and 94% for the *Rr*Nit-FxL1-*Rr*GroEL chimera; although the initial nitrilase activity was reduced by 32–47%. Analyzing the SDS-PAGE results by using Quantity-One software v.4.6.2 (Bio-Rad Laboratories, Hercules, CA, USA), the relative content of fusion protein *Rr*Nit-FxL1-*Rr*GroES, *Rr*Nit-FxL1-*Rr*GroEL and *Rr*Nit-FxL1-*Rr*GroEL2 in total soluble proteins was 51%, 14%, and 24%, respectively (Figure S2). The relative content of unmodified nitrilase in total soluble proteins was 38%. The decrease of expression level in *Rr*Nit-FxL1-*Rr*GroEL and *Rr*Nit-FxL1-*Rr*GroEL2 may be part of the reasons for their activity loss. *Rr*Nit-FxL1-*Rr*GroES with higher expression did not show enhanced nitrilase activity either and the reason may be improper length of the linker. In addition, the residual activity ratio of nitrilase fusion with *Rr*Hsp16 (16 kDa) [24] or rTHS (60 kDa) [27] was no more than 70%, while the residual activity ratio of nitrilase fusion with *Rr*GroES or *Rr*GroEL was over 80% (Figure S3). Therefore, it is reasonable to say that the thermal stability enhancement effects of fusion nitrilase are specific to *Rr*GroES and *Rr*GroEL. *Rr*GroEL2 did not show obvious superiority in either activity or thermal stability improvement of nitrilase compared with *Rr*GroEL, regardless of whether co-expression or fusion-expression was applied. Therefore, in subsequent studies, *Rr*GroEL2 was not further evaluated, and fusion expression of *Rr*GroES and *Rr*GroEL with nitrilase was specifically highlighted for linker optimization.

### 2.2. Effect of Linker Length on the Thermal Stability of RrNit-RrGroES/RrGroEL Fusion Chimeras in E. coli

**Figure 2:**
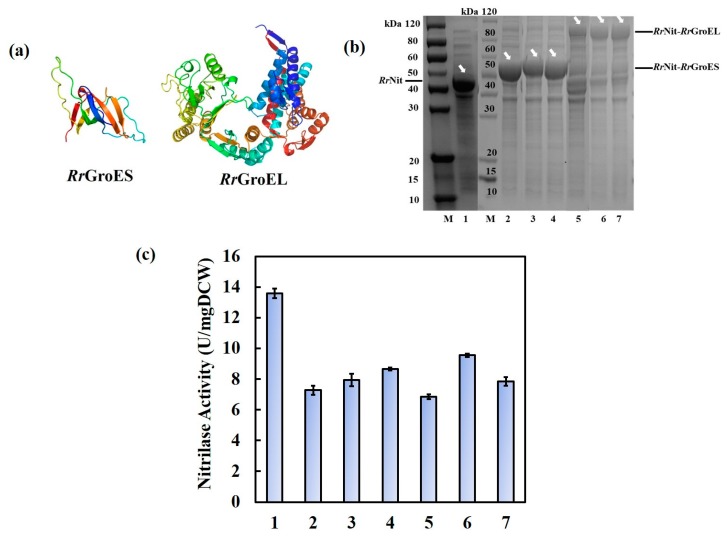
Effect of linker length on the fusion proteins. (**a**) Speculated structure of *Rr*GroES and *Rr*GroES using the online server SWISS-MODEL (https://swissmodel.expasy.org/); (**b**) SDS-PAGE analysis of *Rr*Nit-*Rr*GroES and *Rr*Nit-*Rr*GroEL fusion proteins. M, protein size marker; lane 1 to lane 7, supernatant of cell lysate of *E. coli* BL21(DE3) recombinant strains (pET28a-*Rr*Nit), (pET28a-*Rr*Nit-FxL1-*Rr*GroES), (pET28a-*Rr*Nit-FxL2-*Rr*GroES), (pET28a-*Rr*Nit-FxL4-*Rr*GroES), (pET28a-*Rr*Nit-FxL1-*Rr*GroEL), (pET28a-*Rr*Nit-FxL2-*Rr*GroEL), or (pET28a-*Rr*Nit-FxL4-*Rr*GroEL). Nitrilase, ~40 kDa; *Rr*Nit-*Rr*GroES fusion proteins, ~50 kDa; *Rr*Nit-*Rr*GroEL fusion proteins, ~100 kDa. (**c**) Nitrilase activity of recombinant *E. coli* BL21(DE3) strains with fusion expression of nitrilase and *Rr*GroES or *Rr*GroEL. Lane 1 to lane 7: (pET28a-*Rr*Nit), (pET28a-*Rr*Nit-FxL1-*Rr*GroES), (pET28a-*Rr*Nit-FxL2-*Rr*GroES), (pET28a-*Rr*Nit-FxL4-*Rr*GroES), (pET28a-*Rr*Nit-FxL1-*Rr*GroEL), (pET28a-*Rr*Nit-FxL2-*Rr*GroEL), and (pET28a-*Rr*Nit-FxL4-*Rr*GroEL), respectively.

*Rr*GroES (~10 kDa) and *Rr*GroEL (~60 kDa) are significantly different in size (as shown in Figure 2a). Thus, it is very interesting to know if the linker length between nitrilase and chaperones should change accordingly with the size of the fusion chimeras. Three flexible (GGGGS)_n_ linkers (*n* = 1, 2, or 4, designated FxL1, FxL2, and FxL4, respectively) [28] were selected to verify the effects of the linker on the fusion proteins; in total, six engineered fusion chimeras were constructed and then compared in recombinant *E. coli*: *Rr*Nit-FxL1-*Rr*GroES, *Rr*Nit-FxL2-*Rr*GroES, *Rr*Nit-FxL4-*Rr*GroES, *Rr*Nit-FxL1-*Rr*GroEL, *Rr*Nit-FxL2-*Rr*GroEL and *Rr*Nit-FxL4-*Rr*GroEL. SDS-PAGE results confirmed that each fusion protein was successfully overexpressed (Figure 2b). Analyzing the SDS-PAGE results by Quantity-One software, after fusion with *Rr*GroES, the relative content of fusion protein *Rr*Nit-FxL1-*Rr*GroES, *Rr*Nit-FxL2-*Rr*GroES, and *Rr*Nit-FxL4-*Rr*GroES in total soluble proteins was 52%, 47%, and 47%, respectively; and the relative content of fusion protein *Rr*Nit-FxL1-*Rr*GroEL, *Rr*Nit-FxL2-*Rr*GroEL, and *Rr*Nit-FxL4-*Rr*GroEL was 14%, 27%, and 20%, respectively. Compared to the unmodified nitrilase control (relative content of 38% in total soluble proteins), the expression of the target protein increased after fusion with *Rr*GroES but decreased after fusion with *Rr*GroEL. Moreover, nitrilase activity results showed that all of the fusion proteins with different linkers reduced the initial enzyme activity, and a longer linker seemed better for both chaperones. In particular, for the large *Rr*GroEL chaperone, the short linker (FxL1) in the *Rr*Nit-FxL1-*Rr*GroEL fusion chimera resulted in the lowest nitrilase activity (as shown in Figure 2c).

**Figure 3:**
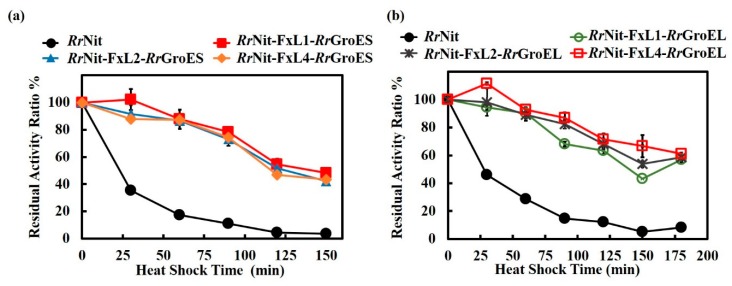
Heat inactivation curves of engineered *E. coli* cells containing chaperone-fused nitrilase with different linkers. (**a**) Nitrilase-*Rr*GroES fusion enzyme in BL21(DE3) (pET28a-*Rr*Nit-FxL1-*Rr*GroES), BL21(DE3) (pET28a-*Rr*Nit-FxL2-*Rr*GroES) and BL21(DE3) (pET28a-*Rr*Nit-FxL4-*Rr*GroES) cells. unmodified nitrilase in BL21(DE3) (pET28a-*Rr*Nit) was used as a control. (**b**), Nitrilase-*Rr*GroEL fusion enzyme in BL21(DE3) (pET28a-*Rr*Nit-FxL1-*Rr*GroEL), BL21(DE3) (pET28a-*Rr*Nit-FxL2-*Rr*GroEL) and BL21(DE3) (pET28a-*Rr*Nit-FxL4-*Rr*GroEL) cells. Unmodified nitrilase in BL21(DE3) (pET28a-*Rr*Nit) was used as a control. Cells were incubated at 50 °C, and the residual nitrilase activity was measured every 30 min. Residual nitrilase activity ratio = Residual nitrilase activity/ Initial activity.

With BL21(DE3) (pET28a-*Rr*Nit) as a control, the thermal stability of the fusion proteins at 50 °C were further compared, and results were summarized, as shown in Figure 3. The thermal stability of all of the fusion proteins was remarkably enhanced, regardless of whether the *Rr*GroES or *Rr*GroEL chaperone was fused with the short or long linker. When the activity of the unmodified nitrilase control dropped to nearly zero, the activity of the *Rr*Nit-*Rr*GroES chimeras remained high, with approximately 50% activity (Figure 3a). For the *Rr*Nit-*Rr*GroEL chimeras, the thermal stability was even better. The *Rr*Nit-FxL4-*Rr*GroEL chimera with the longest linker FxL4 retained approximately 70% activity after 180 min of 50 °C heat shock (Figure 3b).

Assuming that inactivation of intracellular nitrilases fits the first-order kinetic model, the apparent inactivation constant *k_d_* and the half-life *t_1/2_* of different recombinant strains under 50 °C were obtained via curve fitting using MATLAB software (Table 1). The *t_1/2_* of *Rr*Nit-*Rr*GroES chimeras with different linkers (FxL1, FxL2 and FxL4) was 5.8-, 4.9-, and 4.8-fold of that of the unmodified nitrilase, respectively; for *Rr*Nit-*Rr*GroEL chimeras with the same linkers, the *t_1/2_* was further enhanced to 6.2-, 8.7-, and 10.6-fold of that of the unmodified nitrilase, respectively. In general, the *Rr*GroEL chaperone enhanced the thermal stability of nitrilase more remarkably than *Rr*GroES did. In addition, the *Rr*Nit-FxL4-*Rr*GroEL and *Rr*Nit-FxL2-*Rr*GroEL chimeras were the most thermally stable fusion enzymes (Table 1). Considering the activity results, we selected FxL4 and FxL2 as the optimal linkers for *Rr*Nit-*Rr*GroES and *Rr*Nit-*Rr*GroEL, respectively, in subsequent studies.

### 2.3. Fusion- and Co-expression of RrNit and RrGroES/RrGroEL in Parental R. ruber TH3

**Figure 4:**
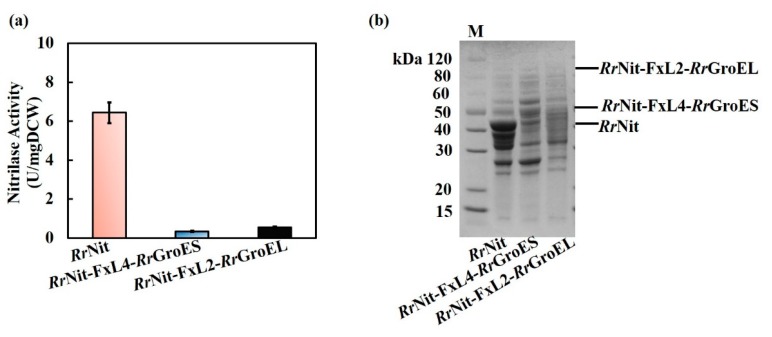
Nitrilase activity and SDS-PAGE results of engineered *R. ruber* TH3 strains. (**a**) Nitrilase activity of engineered *R. ruber* TH3 strains; (**b**) SDS-PAGE results of engineered *R. ruber* TH3 strains. M, Protein size marker; *Rr*Nit, supernatant of cell lysate of *R. ruber* TH3 (pNV18.1-P*ami*-*Rr*Nit); *Rr*Nit-FxL4-*Rr*GroES, supernatant of cell lysate of *R. rube*r TH3 (pNV18.1-P*ami*-*Rr*Nit-FxL4-*Rr*GroES); *Rr*Nit-FxL2-RrGroEL, supernatant of cell lysate of *R. ruber* TH3 (pNV18.1-P*ami*-*Rr*Nit-FxL2-*Rr*GroEL). Nitrilase, ~40 kDa; *Rr*Nit-FxL4-RrGroES fusion protein, ~50 kDa; *Rr*Nit-FxL4-RrGroEL2 fusion protein, ~100 kDa.

Fusion expression of *Rr*GroES/*Rr*GroEL with nitrilase remarkably improved the enzyme thermal stability in *E. coli*. Whether these results were reproducible in the parental strain of the chaperones, *R. ruber*, was of great interest. Thus, the fusion chimeras *Rr*Nit-FxL4-*Rr*GroES and *Rr*Nit-FxL2-*Rr*GroEL were overexpressed in *R. ruber* TH3 using a plasmid vector. Intracellular nitrilase activity was measured, and the results are compared in Figure 4. With respect to the strain harboring unmodified nitrilase, both strains harboring fusion chimeras lost over 90% nitrilase activity, although the finally reached optical density results were approximately the same (approximately OD460 = 50). In Figure 4b, the target fusion protein band of *Rr*Nit-FxL4-*Rr*GroES is shown but relatively weak; and for the *Rr*Nit-FxL2-*Rr*GroEL, the target band can hardly be observed. Therefore, we predicted that the activity loss of the *R. ruber* strains harboring fusion proteins was probably caused by problems in both expression and folding level. But the exact reason remains unknown.

**Figure 5:**
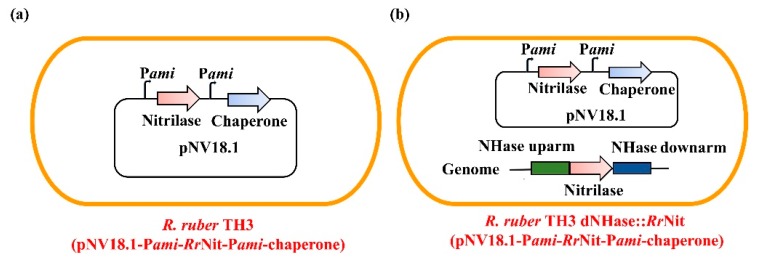
Co-expression design of nitrilase and *Rr*GroES/*Rr*GroEL in *R. ruber* TH3. To improve the expression level, the nitrilase gene was either driven by the strong inducible promoter P*ami* on plasmid pNV18.1, or inserted into the genome-site of NHase (nitrile hydratase) gene. (**a**) plasmid-only expression strategy; (**b**) genome plus plasmid expression strategy.

Since the fusion expression strategy did not work in the parental strain of the chaperones, co-expression of *Rr*GroES/*Rr*GroEL with nitrilase was again tested in *R. ruber* TH3. Two different co-expression strategies were designed and performed: (1) the nitrilase and chaperone genes were only carried with a plasmid vector or (2) the nitrilase gene was inserted into the genome, while a plasmid carrying the nitrilase gene and a chaperone gene was overexpressed, as shown in Figure 5. For the plasmid-only expression strategy, the strong inducible promoter P*ami* [29] was introduced to separately drive nitrilase and *Rr*GroES/*Rr*GroEL expression (Figure 5a). For the genome-plus-plasmid strategy, inspired by the high transcription level of NHase genes in the native *R. ruber* (The FPKM value of NHase gene is up to 1.7 × 10^5^, which is approximately 70-fold of that of the amidase-1 gene which has the second highest transcription level. FPKM, fragments per million fragments mapped, calculated by the formula: Transcript mapped fragments/(Total mapped fragments × Transcript length)) [26], substitution of NHase gene with the nitrilase gene was proposed and accomplished using the novel CRISPR/Cas9 genome editing tool [23], coupled with overexpression of nitrilase and *Rr*GroES/ *Rr*GroES on plasmid (Figure 5b).

**Figure 6:**
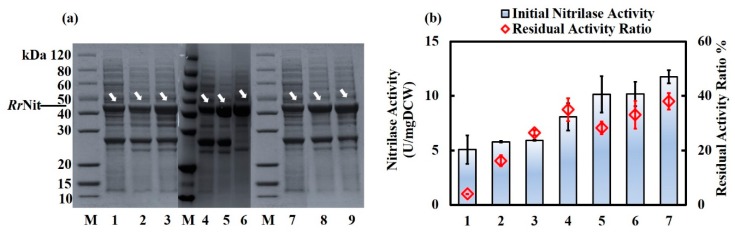
SDS-PAGE results, intracellular nitrilase activity and residual activity ratio results of nitrilase-*Rr*GroEL/*Rr*GroES co-expression strategies in *R. ruber* TH3 cells. (**a**) SDS-PAGE results of the engineered *R. ruber* strains. M, Protein size marker; Lane 1-lane 3, supernatant of cell lysate of TH3 (pNV18.1-P*ami*-*Rr*Nit), TH3 (pNV18.1-P*ami*-*Rr*Nit-P*ami*-*Rr*GroES) and TH3 (pNV18.1-P*ami*-*Rr*Nit-P*ami*-*Rr*GroEL), respectively; Lane 4-lane 6, supernatant of cell lysate of TH3 (pNV18.1-P*ami*-*Rr*Nit), TH3 dNHase::*Rr*Nit and TH3 dNHase::*Rr*Nit (pNV18.1-P*ami*-*Rr*Nit), respectively; Lane 7-lane 9, supernatant of cell lysate of TH3 (pNV18.1-P*ami*-*Rr*Nit), TH3 dNHase::*Rr*Nit (pNV18.1-P*ami*-*Rr*Nit-P*ami*-*Rr*GroES) andTH3 dNHase::*Rr*Nit (pNV18.1-P*ami*-*Rr*Nit-P*ami*-*Rr*GroEL), respectively. Nitrilase, ~40 kDa; (**b**) intracellular nitrilase activity and residual activity ratio results of engineered *R. ruber* strains. 1, the engineered strain *R. ruber* TH3 (pNV18.1-P*ami*-*Rr*Nit); 2, the engineered strain TH3 (pNV18.1-P*ami*-*Rr*Nit-P*ami*-*Rr*GroES); 3, TH3 (pNV18.1-P*ami*-*Rr*Nit-P*ami*-*Rr*GroEL); 4, the engineered strain TH3 dNHase::*Rr*Nit; 5, the engineered strain TH3 dNHase::*Rr*Nit (pNV18.1-P*ami*-*Rr*Nit); 6, the engineered strain TH3 dNHase::*Rr*Nit (pNV18.1-P*ami*-*Rr*Nit-P*ami*-*Rr*GroES); 7, the engineered strain TH3 dNHase::*Rr*Nit (pNV18.1-P*ami*-*Rr*Nit-P*ami*-*Rr*GroEL). The residual activity was measured after 50 °C incubation for 2 h. Residual activity ratio = Residual nitrilase activity/ Initial nitrilase activity.

When nitrilase was only expressed with a plasmid, three engineered strains were constructed: *R. ruber* TH3 (pNV18.1-P*ami*-*Rr*Nit), TH3 (pNV18.1-P*ami*-*Rr*Nit-P*ami*-*Rr*GroES), and TH3 (pNV18.1-P*ami*-*Rr*Nit-P*ami*-*Rr*GroEL). For the genome-plus-plasmid expression strategy, the engineered strain *R. ruber* TH3 dNHase::*Rr*Nit carrying the genome-inserted nitrilase gene in place of the NHase gene site was first constructed, as illustrated in Figure S4. Verification of NHase knockout and nitrilase insertion is shown in Figure S5. Next, using this novel strain as a new host, the engineered strains TH3 dNHase::*Rr*Nit (pNV18.1-P*ami*-*Rr*Nit), TH3 dNHase::*Rr*Nit (pNV18.1-P*ami*-*Rr*Nit-P*ami*-*Rr*GroES), and TH3 dNHase::*Rr*Nit (pNV18.1-P*ami*-*Rr*Nit-P*ami*-*Rr*GroEL) were further obtained after transformation with the appropriate plasmid (Figure S6). SDS-PAGE results showed that nitrilase were successfully overexpressed in the engineered strains, as shown in Figure 6a. Analyzing the SDS-PAGE results by Quantity-One software, the relative content of nitrilase in total soluble proteins was approximately 40% in four strains with nitrilase gene integration in the genome. The relative content of nitrilase in total soluble proteins was ~30% in strain TH3 (pNV18.1-P*ami*-*Rr*Nit-P*ami*-*Rr*GroES) or TH3 (pNV18.1-P*ami*-*Rr*Nit-P*ami*-*Rr*GroEL). Compare to the control *R. ruber* TH3 (pNV18.1-P*ami*-*Rr*Nit) (relative content of 23% in total soluble proteins), the expressions of nitrilase in all recombinant strains were enhanced. Nitrilase activity and thermal stability were measured, and the results are summarized in Figure 6b. Interestingly, the engineered strains harboring the nitrilase gene in the genome at the site of NHase gene showed very high nitrilase activity (1.6- to 2.3-fold of that of plasmid-only expression). The strain TH3 dNHase::*Rr*Nit harboring the nitrilase integration in the genome showed enhanced nitrilase activity and nitrilase thermal activity, 1.6- and 8.7-fold of that of the control TH3 (pNV18.1-P*ami*-*Rr*Nit), respectively. The nitrilase activity and nitrilase thermal stability of the strain TH3 dNHase::*Rr*Nit (pNV18.1-P*ami*-*Rr*Nit), which was constructed by overexpressing nitrilase with a plasmid in TH3 dNHase::*Rr*Nit, was increased by 1.0- and 6.0-fold compared to the control TH3 (pNV18.1-P*ami*-*Rr*Nit), respectively. Furthermore, the engineered strains TH3 dNHase::*Rr*Nit (pNV18.1-P*ami*-*Rr*Nit-P*ami*-*Rr*GroEL) and TH3 dNHase::*Rr*Nit (pNV18.1-P*ami*-*Rr*Nit-P*ami*-*Rr*GroES) in which the *Rr*GroEL or *Rr*GroES chaperone was overexpressed showed significantly enhanced nitrilase thermal stability (8.4- and 7.3-fold, respectively); the nitrilase activity of the strains was increased by 1.0- and 1.3-fold, respectively. Again, the stabilizing effect of *Rr*GroEL was more significant than that of *Rr*GroES when co-expressed with nitrilase in the native *R. ruber*, similar to the results of fusion-expression in *E. coli*. Co-enhanced enzyme activity and thermostability are highly valuable for bioindustrial applications.

## 3. Discussion

Chaperones play important roles in assisting correct intracellular protein folding and protection of proteins under stress conditions [20]. For example, stress-protective proteins, such as GroEL from *Bacillus subtilis*, induced under heat pretreatment, might protect the cell from heat damage [30]. The GroES-GroEL chaperonin from *Thermus thermophilus* can aid in refolding of various denatured proteins [31]. Different bacterial species have developed their own chaperone systems according to the stresses they encounter and the different mechanisms of chaperone function against thermotolerance [32]. In general, GroEL can bind proteins and prevent them from aggregation under heat stress [33]. GroEL can also assist and facilitate correct folding of a variety of newly synthesized proteins to achieve their active forms [15]. GroEL and GroES from *E. coli* and thermophilic strains have been well studied previously [20,21,31,34]. Chaperones are important in nitrilase folding because nitrilase from *Gibberella moniliformis* or *Penicillium marneffei* was found to co-purify with chaperones such as GroEL [18]. In 1999, Almatawah et al. found that GroEL was binding strongly to nitrilase in a thermophilic strain *Bacillus pallidus* and the half-life of nitrilase-GroEL complex was up to 8.4 h at 50 °C, which indicated GroEL may play an important role in stabilizing nitrilase at high temperature [35]. In this study, we highlighted the novel chaperones *Rr*GroEL and *Rr*GroES in *R. ruber*, a nontypical actinomycete with high organic solvent tolerance.

Enzymes are considered good candidate catalysts for industrial chemical production owing to their high activity, selectivity and sustainability. Typically, enzymes function well under mild conditions but are prone to inactivation in unsuitable environments, such as high temperature. Therefore, enzyme thermal stability is very important in various industrial applications [36]. For example, the thermal stability of starch-converting enzymes is of great importance because it can avoid problems such as a long wait time after gelatinization with heat [9]. High temperatures are also required for xylanase to remove residual lignin from pulp [9,37,38]. Lipases are widely used in many bioconversion reactions in the food industry, pharmaceutical industry and for other applications. Most industrial lipase-involved processes are conducted at reaction temperatures over 45 °C [9,39]. The low stability of nitrilase is thought to be one of the reasons limiting its industrial application [8].

To enhance the thermal stability of enzymes, various methods have been proposed in the literature at the genetic engineering level, such as random mutagenesis and rational design [11,12]. However, random mutagenesis usually requires many subsequent screening processes, and rational design always requires knowledge of the enzyme structure [40]. Hence, a chaperone fusion/co-expression strategy is a very simple but efficient choice for enhancing enzyme stability regardless of whether the protein structure is known. Different from previous studies that primarily focused on the coupling functions of GroEL-GroES, in this work, we highlighted the effect of a single *Rr*GroEL or *Rr*GroES chaperone.

Using nitrilase from *R. rhodochrous* tg1-A6 as the model enzyme, fusion expression and co-expression of *Rr*GroEL/*Rr*GroES with nitrilase were first assessed in *E. coli* host. We found that *Rr*GroEL/*Rr*GroES fusion expression can dramatically enhance the thermal stability of nitrilase up to 8.7- to 10.6-fold. Nitrilase is prone to form a multimer which is composed of identical subunits [41]. The nitrilase from *Bacillus pallidus*, which is a thermophilic bacterium, was purified as a complex with a putative GroEL (total molecular weight: 600 kDa) and had a subunit molecular weight of 41 kDa; and the half-life of purified nitrilase-GroEL was up to 8.4 h at 50 °C [35]. Hence, the positive effect of GroEL on nitrilase sounds a universal phenomenon. After fusion with either *Rr*GroES or *Rr*GroEL, engineered *E. coli* strains harboring fusion chimeras showed reduced nitrilase activity, which may be due to either the lower expression level of fusion chimera or improper folding of a large protein. However, the exact reason remains unknown yet. In addition, we found that the small assistant chaperone *Rr*GroES can also act as an effective fusion tag for thermal stability enhancement, similar to *Rr*GroEL, although the improvement effect is a bit weaker than that of GroEL. In our previous study, *Rr*GroES fusion with NHase increased both NHase activity by 26.5% and residual activity after heat shock by 1.6-fold as well [14]. This special result is only reported with *Rr*GroES so far. Other GroES chaperones, such as *Ec*GroES, should be also tested in future studies, to identify if *Rr*GroES is unique and propose a probable explanation for this. Additionally, considering that the size of *Rr*GroES is only ~10 kDa, significantly smaller than *Rr*GroEL (~60 kDa), applying *Rr*GroES as a universal stabilizing tag for different industrial enzymes is not only feasible and but valuable.

The effect of the linker on fusion protein activity was also investigated due to the important role of linkers in connecting proteins and helping the inter-domain interactions of fusion chimeras [42]. The widely used flexible linker (GGGGS)_n_ was used in this study. Similar to previous results [43], a relatively longer linker was required for the larger *Rr*GroEL (~60 kDa) fusion tag. A linker that is too short will result in tertiary structure disturbance of the two proteins, thereby reducing the final apparent activity.

*Rhodococcus* spp. are important industrial strains. In particular, *R. ruber* whole-cell biocatalysts are highly promising for production of diverse valuable chemicals [5,22]. Therefore, the host effect of *Rr*GroEL/*Rr*GroES fusion expression was further assessed with *R. ruber*, the parental chaperone strain, as the host. We found that the *Rr*GroEL/*Rr*GroES chaperones cannot be used as a fusion tag of the enzyme-chimeras in the parental host *R. ruber*. For the case of *Rr*GroEL/*Rr*GroES-nitrilase fusion chimeras expressed in *R. ruber*, both intracellular fusion enzymes lost over 90% nitrilase activity. Co-expression with chaperones strategy is often used to improve the overexpression of intracellular proteins. Petříčková et al. found co-expression with GroEL-GroES improve specific activity of nitrilases from *Gibberella moniliformis* and *Penicillium marneffei* [18]. A co-expression strategy was then re-proposed by insertion of the nitrilase gene in the genome of *R. ruber*, with simultaneous overexpression of *Rr*GroEL or *Rr*GroES via plasmid, two novel engineered strains (TH3 dNHase::*Rr*Nit (pNV18.1-P*ami*-*Rr*Nit-P*ami*-*Rr*GroES) and TH3 dNHase::*Rr*Nit (pNV18.1-P*ami*-*Rr*Nit-P*ami*-*Rr*GroEL)) were obtained, and their nitrilase activity and thermal stability were both remarkably enhanced. Compared with *Rr*GroES, the *Rr*GroEL and nitrilase co-expressing mutant showed the best performance: nitrilase activity was increased by 1.3-fold (up to 11.8 U/mgDCW), and thermal stability was enhanced by 8.4-fold compared with the control strain TH3 (pNV18.1-P*ami*-*Rr*Nit). This novel engineered strain is promising for industrial production of acrylic acid.

In addition, only co-expression or fusion expression of nitrilase with one single chaperone was tested in this work. This is in light of our previous study on the function of *Rr*GroEL2 and/or *Rr*GroES toward nitrile hydratase, in which the independent RrGroEL2 has a better stabilizing effect than the coupled *Rr*GroES + *Rr*GroEL2 on nitrile hydratase at 90 °C [14]. But whether the synergic effect between *Rr*GroES and *Rr*GroEL exists or not on different enzymes in different hosts still requires further verification in the future.

In sum, this study presents a new, highly valuable method to enhance the important performance of industrial whole-cell biocatalysts—simply through fusion with (in an *E. coli* host) or co-expression with the novel chaperone *Rr*GroEL (in *R. ruber*).

## 4. Materials and Methods

### 4.1. Plasmids, Strains and Chemicals

All strains and plasmids used in this study are listed in Table S1. Plasmid pET28a (with T7 promoter) (Merck KGaA, Darmstadt, Germany) and pNV18.1 (National Institute of Infectious Diseases, Tokyo, Japan) were used as protein expression vectors. The nitrilase gene (EF467367.1) was from a mutant strain of *R. rhodochrous* tg1-A6 [6]. The *Rr*GroES, *Rr*GroEL and *Rr*GroEL2 genes were cloned from *R. ruber* TH. *E. coli* Top10 for recombinant plasmid construction and cloning was purchased from Biomed (Beijing, China). *E. coli* BL21(DE3) was used to express target proteins. *R. ruber* TH3 is the amidase-deleted mutant of *R. ruber* TH. It carries the same chaperones *Rr*GroES, *Rr*GroEL and *Rr*GroEL2 with strain TH. *R. ruber* TH3 also has a native nitrilase gene, but the enzyme activity of the natural nitrilase is too low to be detected.

Taq DNA polymerase, Phanta DNA polymerase and T4 DNA ligase were purchased from Vazyme (Nanjing, China). Restriction endonucleases were purchased from Takara (Shiga, Japan). All PCR primers are listed in Table S2.

### 4.2. Construction of the Recombinant Strains

Target genes were amplified using Phanta DNA polymerase and standard PCR protocols. The DNA samples were obtained after agarose gel electrophoresis and gel extraction. Enzyme digestion, DNA ligation and plasmid transformation were performed according to standard procedures [44] and the relevant manufacturer’s instructions.

The BL21(DE3) (pET28a-*Rr*Nit-FxL1-*Rr*GroES/*Rr*GroEL/GroEL2) strains expressed *Rr*Nit-*Rr*GroES/*Rr*GroEL/*Rr*GroEL2 chimeras with the T7 promoter. These chaperones were fused to the C-terminus of the nitrilase gene by a (GGGGS)_1_ linker (FxL1) via overlap PCR. The BL21(DE3) (pET28a-*Rr*Nit-FxL1/FxL2/FxL4/-*Rr*GroES) and BL21(DE3) (pET28a-*Rr*Nit-FxL1/FxL2/FxL4/-*Rr*GroEL) strains with different linkers were constructed in a similar manner.

BL21(DE3) (pET28a-*Rr*Nit+*Rr*GroES) co-expressed *Rr*Nit and the chaperone *Rr*GroES through a single T7 promoter. The ribosome binding site (RBS: TTTGTTTAACTTTAAGAAGGAGA) was inserted after the C-terminus of the nitrilase gene. BL21(DE3) (pET28a-*Rr*Nit+*Rr*GroEL) and BL21(DE3) (pET28a-*Rr*Nit+*Rr*GroEL2) were constructed in a similar manner.

In situ substitution of NHase with nitrilase in the *R. ruber* TH3 strain was accomplished by following the CRISPR/Cas9 genome editing method for *R. ruber* [23]. *R. ruber* TH3 was transformed with pNV18.1-Pa2-Cas9 and then pRCTc-Pa2-Che9c60&61 [23]. *R. ruber* TH3 (Cas9+Che9c60&61) with pNV18.1-Pa2-Cas9 and pRCTc-Pa2-Che9c60&61 was subsequently transformed with pBNVCm-sgRNA and linear donor dsDNA [23]. Cells were spread on a plate with 25 μg/mL kanamycin, 6 μg/mL tetracycline and 5 μg/mL chloramphenicol to obtain target strains.

### 4.3. Culture Conditions for the Recombinant Strains

*E. coli* Top 10 and BL21(DE3) were grown in Luria-Bertani (LB) medium (tryptone, 10 g L^−1^; yeast extract, 5 g·L^−1^; and NaCl, 10 g·L^−1^). *E. coli* BL21(DE3) was cultivated in 300 mL shaking flasks containing 50 mL LB medium at 37 °C with 1% inoculation of the seed medium for 12 h. When the OD_600_ of the culture medium reached 2.0, 0.02 M lactose was added as an inducer. The culture was moved to a 28 °C shaker and cultivated for 7 h. Antibiotic was added if necessary (kanamycin 50 μg mL^−1^).

Seed medium (per liter: glucose 20 g; yeast extract 1 g; tryptone 7 g; K_2_HPO_4_·3H_2_O 0.5 g; KH_2_PO_4_ 0.5 g; MgSO_4_·7H_2_O 0.5 g; monosodium glutamate 1 g; pH 7.5) and fermentation medium (per liter: glucose 30 g; yeast extract 7–8 g; urea 10 g; K_2_HPO_4_·3H_2_O 0.866 g; KH_2_PO_4_ 2.28 g; MgSO_4_·7H_2_O 1 g; monosodium glutamate 1 g; pH 7.5) were used for *R. ruber* cultivation. *R. ruber* cells were cultivated for 48 h at 28 °C with an initial OD_460_ = 3.0 after seeding. Antibiotic was added if necessary (kanamycin 25 μg mL^−1^).

### 4.4. Assay of Nitrilase Activity

Nitrilase activity was determined in a 1020 μL reaction mixture at 28 °C. For *E. coli* cells, 20 μL acrylonitrile was added into 1 mL resuspended cells with OD_600_ = 5.0 in 0.1 M PBS buffer (pH 7.5). For *R. ruber* cells, 20 μL acrylonitrile was added into 1 mL of a mixture containing 200 μL resuspended cells with OD_460_ = 40.0 in 0.1 M PBS and 800 μL of 0.1 M PBS buffer (pH 7.5) [26]. The reaction was terminated by adding 100 μL of 3 M HCl. The mixture was centrifuged at 13,000 rpm for one minute, and the supernatant was used to analyze the ammonium acrylate concentration via gas chromatography (Abel Industries Canada Ltd., Vancouver BC Canada, AB-INOWAX 30 m × 0.25 mm × 0.25 μm). Acetamide was used as the internal standard. The GC operation conditions were as follows: a TRACE 1300 GC device was equipped with a polyethylene glycol polymer capillary column (30 m × 0.25 mm × 0.25 μm) and a flame ionization detector—the injection and detector temperatures were 260 °C, while the column temperature was 190 °C.

One unit (U) of nitrilase activity was defined as the quantity of ammonium acrylate (μmol) that was catalyzed by 1 mg DCW (DCW, dry cell weight) cell suspension in one minute.

### 4.5. In-cell Nitrilase Stability Evaluation

The fermentation broth was centrifuged at 8000 rpm and 4 °C for 10 min, and the supernatant was removed. After centrifugation, the cells were resuspended to an OD_600_ value of 5.0 (*E. coli* cells) or an OD_460_ value of 40.0 (*R. ruber* cells) in 0.1 M PBS. Initial enzyme activity (*v_0_*) was measured at 28 °C. Resting cells were treated at 50 °C for specific time. Then, the cells were placed at 28 °C for 10 min to cool. The residual activity (*v*) was measured next. Assuming that inactivation of the intracellular enzymes after heat shock follows the first-order model, the parent inactivation constant *k_d_* and the half-life t_1/2_ were obtained by data fitting via MATLAB according to the formula ln(*v*/*v* = −*k_d_* *t and *t_1/2_* = −ln2/ *k_d_*.

## Figures and Tables

**Table 1:** Apparent kinetic parameters for thermal inactivation of cell catalysts harboring nitrilase and *Rr*GroES/*Rr*GroEL chimeras at 50 °C.

Cell Catalysts.	*k_d_* (min^−1^)	*t_1/2_* (min)
BL21(DE3) (pET28a-*Rr*Nit)	0.02453 ± 0.00057	28.27 ± 0.66
BL21(DE3) (pET28a-*Rr*Nit-FxL1-*Rr*GroES)	0.00445 ± 0.00039	156.81 ± 13.90
BL21(DE3) (pET28a-*Rr*Nit-FxL2-*Rr*GroES)	0.00505 ± 0.00051	138.67 ± 14.00
BL21(DE3) (pET28a-*Rr*Nit-FxL4-*Rr*GroES)	0.00514 ± 0.00019	135.04 ± 4.94
BL21(DE3) (pET28a-*Rr*Nit-FxL1-*Rr*GroEL)	0.00368 ± 0.00053	92.34 ± 27.70
BL21(DE3) (pET28a-*Rr*Nit-FxL2-*Rr*GroEL)	0.00286 ± 0.00036	246.26 ± 31.00
BL21(DE3) (pET28a-*Rr*Nit-FxL4-*Rr*GroEL)	0.00230 ± 0.00005	301.57 ± 7.87

## Supplementary Materials

The following are available online, Figure S1: Transcription level changes of *R. ruber* chaperones *Rr*GroES, *Rr*GroEL and *Rr*GroEL2; Figure S2: SDS-PAGE results of fusion chimeras; Figure S3: Nitrilase activity and the residual activity ratio of nitrilase in the *E. coli* BL21(DE3) recombinant strains (pET28a-*Rr*Nit), (pET28a-*Rr*Nit-FxL1-*Rr*Hsp16) and (pET28a-*Rr*Nit-FxL1-rTHS); Figure S4: Substitution of NHase with nitrilase using CRISPR/Cas9 tools in the genome of *R. ruber* TH3; Figure S5: DNA electrophoresis results to verify the successful deletion of NHase and insertion of nitrilase; Figure S6: CRISPR/Cas9 systems for *R. ruber* and the experimental process for constructing the engineered strains *R. ruber* TH3 dNHase::*Rr*Nit, TH3 dNHase::*Rr*Nit (pNV18.1-P*ami*-*Rr*Nit-P*ami*-*Rr*GroES) and TH3 dNHase::*Rr*Nit (pNV18.1-P*ami*-*Rr*Nit-P*ami*-*Rr*GroEL); Table S1:Plasmids and strains used in this study; Table S2: PCR primers used in this study.
