# Supplementary material for: Novel Chaperones RrGroEL and RrGroES for Activity and Stability Enhancement of Nitrilase in Escherichia coli and Rhodococcus ruber
Source: Molecules. 2020 Feb 24;25(4):1002. doi: 10.3390/molecules25041002 (PMC7070999; doi:10.3390/molecules25041002)
Supplement: Supplementary file 1 [file molecules-25-01002-s001.pdf]

Article

Novel chaperones *Rr*GroEL and *Rr*GroES for activity and stability enhancement of nitrilase in *Escherichia coli* and *Rhodococcus ruber*

Chunmeng Xu#[1,2], Lingjun Tang#[1,2], Youxiang Liang[1,2], Song Jiao, Huimin Yu [1,2,3,*] and Hui Luo[4]

[1] Key Laboratory of Industrial Biocatalysis (Tsinghua University), the Ministry of Education, Beijing 100084, P. R. China
[2] Department of Chemical Engineering, Tsinghua University, Beijing 100084, P. R. China
[3] Center for Synthetic and Systems Biology, Tsinghua University, Beijing 100084, P. R. China
[4] Department of Biological Science and Engineering, University of Science and Technology Beijing, Beijing 100083, China
[#] These two authors contributed equally to this work.
* Correspondence: yuhm@tsinghua.edu.cn (Huimin Yu); Tel.: 86-10-62795492

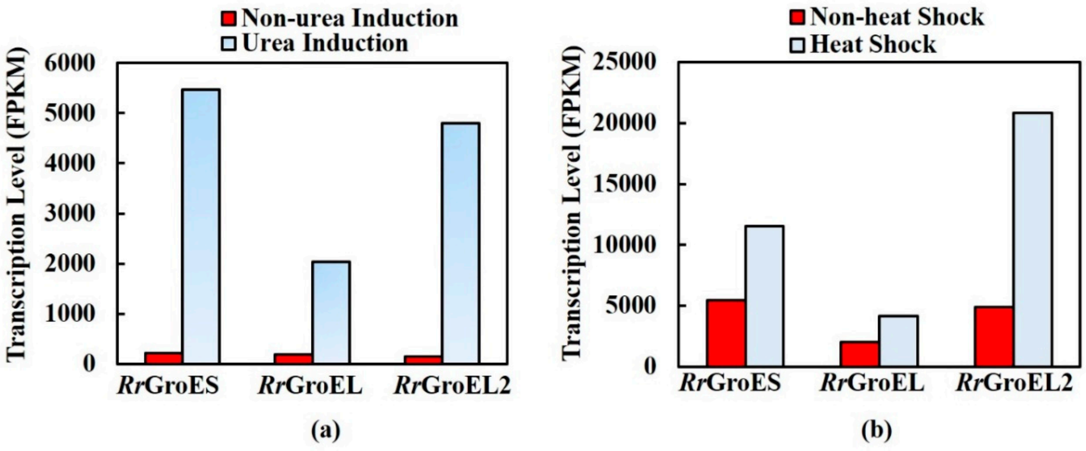

Figure S1. Transcription level changes of *R. ruber* chaperones *Rr*GroES, *Rr*GroEL and *Rr*GroEL2[1]. (a) Transcription level under non-urea induction and urea induction conditions; (b) Transcription level under non-heat shock and heat shock conditions. FPKM, fragments per million fragments mapped, calculated by the formula: Transcript mapped fragments/(Total mapped fragments × Transcript length) .

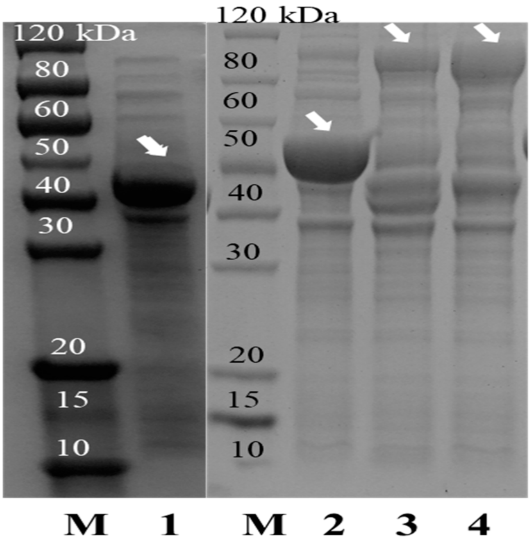

Figure S2. SDS-PAGE results of fusion chimeras. M, protein size marker; Lane 1-lane 4, supernatant of cell lysate of recombinant strains BL21(DE3) (pET-T7-Nit), recombinant strains BL21(DE3) (pET-T7-Nit-FxL1-*Rr*GroES), recombinant strains BL21(DE3) (pET-T7-Nit FxL1-*Rr*GroEL) and recombinant strains BL21(DE3) (pET-T7-Nit-FxL1-*Rr*GroEL2). Nitrilase, ~40 kDa; Nit-FxL1-*Rr*GroEL fusion protein, ~100 kDa; Nit-FxL1-*Rr*GroEL2 fusion protein, ~100 kDa.

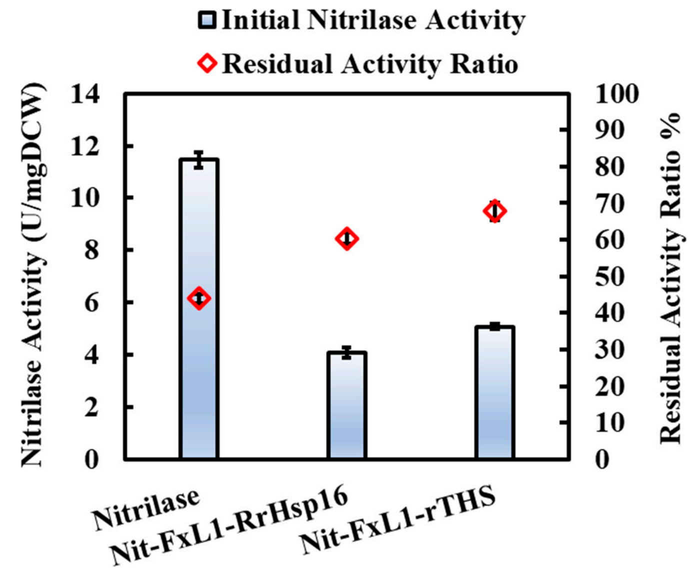

Figure S3. Nitrilase activity and the residual activity ratio of the *E. coli* recombinant strains BL21(DE3) (pET-T7-Nit), BL21(DE3) (pET-T7-Nit-FxL1-*Rr*Hsp16) and BL21(DE3) (pET-T7-Nit-FxL1-rTHS). Residual activity ratio = Residual nitrilase activity/ Initial nitrilase activity.

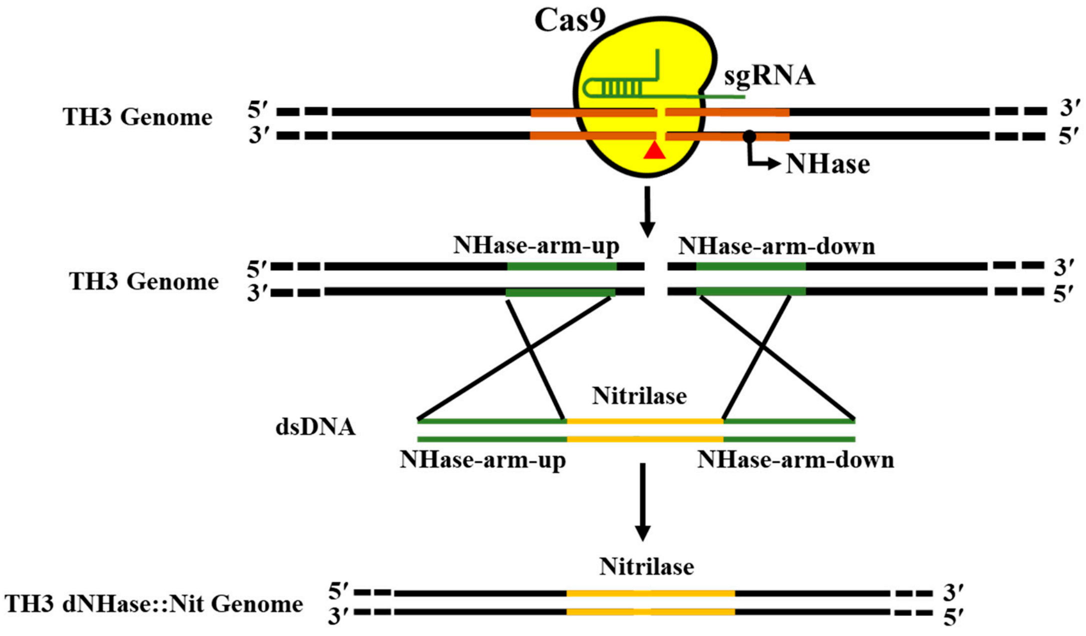

Figure S4. Substitution of NHase with nitrilase using CRISPR/Cas9 tools [2] in the genome of *R. ruber* TH3.

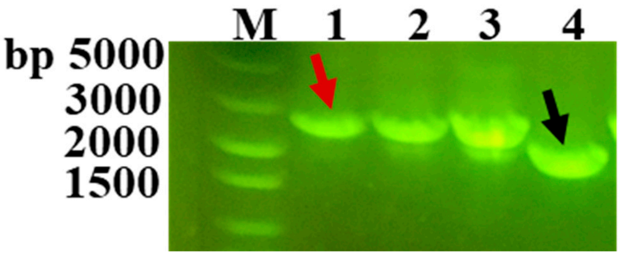

Figure S5. DNA electrophoresis results to verify the successful deletion of NHase and insertion of nitrilase. Lane 1, marker; lane 2-3, *R. ruber* TH3 (~2400bp); lane 4, *R. ruber* TH3 dNHase::Nit (~2200 bp).

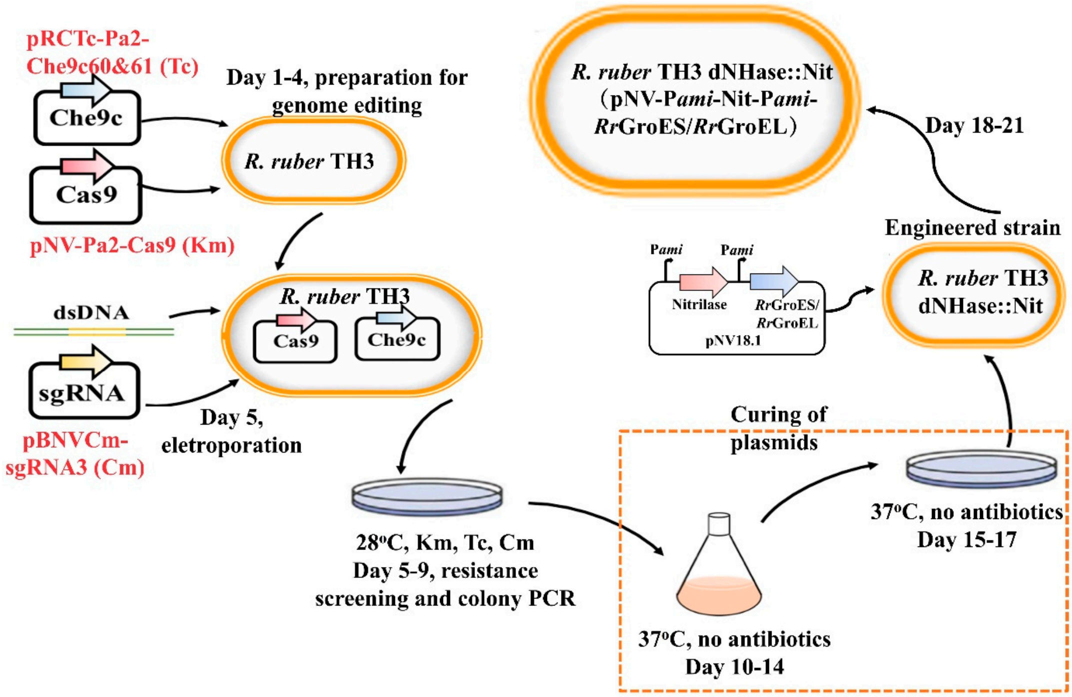

Figure S6. CRISPR/Cas9 systems for *R. ruber* and the experimental process for constructing the engineered strains *R. ruber* TH3 dNHase::Nit, *R. ruber* TH3 dNHase::Nit (pNV-P*ami*-Nit), TH3 dNHase::Nit (pNV-P*ami*-Nit-P*ami*-*Rr*GroES) and TH3 dNHase::Nit (pNV-P*ami*-Nit-P*ami*-*Rr*GroEL).

Table S1. Plasmids and strains used in this study

Strains/plasmids	Relevant genotypes	source
**Plasmids**		
pET28a	Parental blank plasmid	Merck KGaA
pET-T7-Nit	With nitrilase (Nit) gene	This study
pET-T7-Nit+*Rr*GroES	With Nit and *Rr*GroES genes,	This study
pET-T7-Nit+*Rr*GroEL	With Nit and *Rr*GroEL genes,	This study
pET-T7-Nit+*Rr*GroEL2	With Nit and *Rr*GroEL2 genes,	This study
pET-T7-Nit-FxL1-*Rr*GroEL2	With Nit and *Rr*GorEL2 genes, flexible linker $(GGGGS)_1$	This study
pET-T7-Nit-FxL1-*Rr*Hsp16	With Nit and *Rr*Hsp16 genes, flexible linker $(GGGGS)_1$	This study
pET-T7-Nit-FxL1-rTHS	With Nit and rTHS genes, flexible linker $(GGGGS)_1$	This study
pET-T7-Nit-FxL1-*Rr*GroES	With Nit and *Rr*GorES genes, flexible linker $(GGGGS)_1$	This study
pET-T7-Nit-FxL2-*Rr*GroES	With Nit and *Rr*GroES genes, flexible linker $(GGGGS)_2$	This study

pET-T7-Nit-FxL4-*Rr*GroES	With Nit and *Rr*GroES genes, flexible linker $(GGGGS)_4$	This study
pET-T7-Nit-FxL1-*Rr*GroEL	With Nit and *Rr*GorEL genes, flexible linker $(GGGGS)_1$	This study
pET-T7-Nit-FxL2-*Rr*GroEL	With Nit and *Rr*GroEL genes, flexible linker $(GGGGS)_2$	This study
pET-T7-Nit-FxL4-*Rr*GroEL	With Nit and *Rr*GroEL genes, flexible linker $(GGGGS)_4$	This study
pNV18.1	*E. coli-Rhodococcus* shuttle vector	[3]
pNV-P*ami*-Nit	With Nit genes, Pami Promotor	This study
pNV-P*ami*-Nit-FxL4-*Rr*GroES	With Nit and *Rr*GroES genes, flexible linker $(GGGGS)_4$	This study
pNV-P*ami*-Nit-FxL2-*Rr*GroEL	With Nit and *Rr*GroEL genes, flexible linker $(GGGGS)_2$	This study
pNV-P*ami*-Nit-P*ami*-*Rr*GroES	With Nit and *Rr*GroES genes, promoted by two separated Pami	This study
pNV-P*ami*-Nit-P*ami*-*Rr*GroEL	With Nit and *Rr*GroEL genes, promoted by two separated Pami	This study
pBNVCm-sgRNA3	sgRNA targeting NHase gene *nhh*, Cm	[2]
pRCTc-Pa2-Che9c60&61	Reombinase genes *che9c60&61*, promoter Pa2, Tc	[2]
pNV-Pa2-Cas9	codon-optimized *cas9*, promoter Pa2, Km	[2]
**Strains**		
Top10	Cloning host	Biomed
BL21(DE3)	Protein expression host	Biomed
BL21(DE3) (pET-T7-Nit)	pET-28a, Nit, T7 promoter	This study
BL21(DE3) (pET-T7-Nit+*Rr*GroES)	Nit and *Rr*GroES co-expression with T7	This study
BL21(DE3) (pET-T7-Nit+*Rr*GroEL)	Nit and *Rr*GroEL co-expression with T7	This study
BL21(DE3) (pET-T7-Nit+*Rr*GroEL2)	Nit and *Rr*GroEL2 co-expression with T7	This study
BL21(DE3) (pET-T7-Nit-FxL1-*Rr*GroEL2)	Nit-*Rr*GroEL2 fusion expression, linker $(GGGGS)_1$	This study
BL21(DE3) (pET-T7-Nit-FxL1-*Rr*Hsp16)	Nit- *Rr*Hsp16 fusion expression, linker $(GGGGS)_1$	This study
BL21(DE3) (pET-T7-Nit-FxL1-rTHS)	Nit- rTHS fusion expression, linker $(GGGGS)_1$	This study
BL21(DE3) (pET-T7-Nit-FxL1-*Rr*GroES)	Nit-*Rr*GroES fusion expression, linker $(GGGGS)_1$	This study
BL21(DE3) (pET-T7-Nit-FxL2-*Rr*GroES)	Nit-*Rr*GroES fusion expression, linker $(GGGGS)_2$	This study
BL21(DE3) (pET-T7-Nit-FxL4-*Rr*GroES)	Nit-*Rr*GroES fusion expression, linker $(GGGGS)_4$	This study
BL21(DE3) (pET-T7-Nit-FxL1-*Rr*GroEL)	Nit-*Rr*GroEL fusion expression, linker $(GGGGS)_1$	This study
BL21(DE3) (pET-T7-Nit-FxL2-*Rr*GroEL)	Nit-*Rr*GroEL fusion expression, linker $(GGGGS)_2$	This study

BL21(DE3) (pET-T7-Nit-FxL4-*Rr*GroEL)	Nit-*Rr*GroEL fusion expression, linker $(GGGGS)_4$	This study
R. ruber TH3	Amidase knockout, parental host	[4]
R. ruber TH3 (pNV-P*ami*-Nit)	Expression of nitrilase	This study
R. ruber TH3 dNHase::Nit	Substitution of NHase gene with nitrilase in genome	This study
R. ruber TH3 dNHase::Nit (pNV-P*ami*-Nit)	Substitution of NHase gene with nitrilase in genome, expression of nitrilase	This study
*R. rube*r TH3 (pNV-P*ami*-Nit-FxL4-*Rr*GroES)	Nit-*Rr*GroES fusion expression, linker $(GGGGS)_4$	This study
*R. rube*r TH3 (pNV-P*ami*-Nit-FxL2-*Rr*GroEL)	Nit-*Rr*GroEL fusion expression, linker $(GGGGS)_2$	This study
R. ruber TH3 (pNV-P*ami*-Nit-P*ami*-*Rr*GroES)	Co-expression of nitrilase and *Rr*GroES	This study
R. ruber TH3 (pNV-P*ami*-Nit-P*ami*-*Rr*GroEL)	Co-expression of nitrilase and *Rr*GroEL	This study
R. ruber TH3 dNHase::Nit (pNV-P*ami*-Nit-P*ami*-*Rr*GroES)	Substitution of NHase gene with nitrilase in genome, co-expression of nitrilase and *Rr*GroES	This study
R. ruber TH3 dNHase::Nit (pNV-P*ami*-Nit-P*ami*-*Rr*GroELS)	Substitution of NHase with nitrilase in genome, co-expression of nitrilase and *Rr*GroES	This study

Table S2. PCR primers used in this study

**Primers**	**Sequence**
NcoI-Nit -S	CATGCCATGGTCGAATACACAAACACATTCAAAGTTG
Nit-RBS-A	GGTATATCTCCTTCTTAAAGTTAAACAAAATTATTTCAGATGG AGGCTGTCGCC
RBS-GroEL2-S	AAATAATTTTGTTTAACTTTAAGAAGGAGATATACCATGGCCA AGATCATCGCGTT
GroEL2-HindIII-A	CCCAAGCTTTCAGAAGTCCATGCCACCCAT
RBS-GroEL-S	AAATAATTTTGTTTAACTTTAAGAAGGAGATATACCATGTCCA AGCAAATCGAGTTCAACGA
GroEL-HindIII-A	CCCAAGCTTCTAGTGAGCGTGACCGTGGC
RBS-GroES-S	AAATAATTTTGTTTAACTTTAAGAAGGAGATATACCATGAACA TCAAGCCGCTCGAGGA
GroES-HindIII-A	CCCAAGCTTTTACTTGGCGACGACGGCC
Nit-FxL1-GroEL2-A	CGCGATGATCTTGGCCATCGAACCACCACCACCTCAGATGGAG GCTGTCGCCC
GroEL2-S	ATGGCCAAGATCATCGCGTTC
GroEL2-fusion-A-HindIII	CCCAAGCTTTCAGAAGTCCATGCCACCC
Nit-FxL1-GroES-A	GAGCGGCTTGATGTTCATCGAACCACCACCACCTCAGATGGAG

	GCTGTCGCCCG
GroES-fusion-S	ATGAACATCAAGCCGCTCGAGG
GroES-fusion-A-HindIII	CCCAAGCTTTTACTTGGCGACGACGGC
Nit-FxL1-GroEL-A	CTCGATTTGCTTGGACATCGAACCACCACCACCTCAGATGGAG GCTGTCGCCCG
GroEL-fusion-S	ATGTCCAAGCAAATCGAGTTCAACG
GroEL-fusion-A-HindIII	CCCAAGCTTCTAGTGAGCGTGACCGTGGC
Nit-FxL2-GroES-A	TGTTCATCGAACCACCACCACCCGAACCACCACCACCTCAGAT GGAGGCTGTCGCCCG
FxL2-GroES-S	GTTCGGGTGGTGGTGGTTCGATGAACATCAAGCCGCTCGA
Nit-FxL4-GroES-A	GCGGCTTGATGTTCATCGAACCACCACCACCCGAACCACCACC ACCCGAACCACCACCACCCGAACCACCACCACCTCAGATGGA GGCTGTCGCCCG
Nit-FxL2-GroEL-A	CTCGATTTGCTTGGACATCGAACCACCACCACCCGAACCACCA CCACCTCAGATGGAGGCTGTCGCCCG
Nit-FxL4-GroEL-A	CTCGATTTGCTTGGACATCGAACCACCACCACCCGAACCACCA CCACCCGAACCACCACCACCCGAACCACCACCACCTCAGATG GAGGCTGTCGCCCG
Pami-XbaI-S	GCTCTAGATGCGGACGGCGGATAC
Pami-PstI-A	TGCACTGCAGCTCCTTAGTGACTCGCCGG
Nit-PstI-S	TGCACTGCAGATGGTCGAATACACAAACACATTCA
GroES-fusion-A-HindIII	CCCAAGCTTTTACTTGGCGACGACGGC
GroEL-fusion-A-HindIII	CCCAAGCTTCTAGTGAGCGTGACCGTGGC
Nit-Pami-A	TCAGATGGAGGCTGTCGCCC
Nit-Pami-S	GGGCGACAGCCTCCATCTGATGCGGACGGCGGATACG
Pami-A	CTCCTTAGTGACTCGCCGGGC
Pami-GroES-S	GCCCGGCGAGTCACTAAGGAGATGAACATCAAGCCGCTCGAG
Pami-GroEL-S	GCCCGGCGAGTCACTAAGGAGATGTCCAAGCAAATCGAGTTC AACG

References:

1. Chen, Y.; Jiao, S.; Wang, M.; Chen, J.; Yu, H., A novel molecular chaperone GroEL2 from *Rhodococcus ruber* and its fusion chimera with nitrile hydratase for co-enhanced activity and stability. *Chem. Eng. Sci.* **2018,** 192, 235-243.

2. Liang, Y.; Jiao, S.; Wang, M.; Yu, H.; Shen, Z., A CRISPR/Cas9-based genome editing system for Rhodococcus ruber TH. *Metab. Eng.* **2020,** 57, 13-22.
3. Chiba, K.; Hoshino, Y.; Ishino, K.; Kogure, T.; Mikami, Y.; Uehara, Y.; Ishikawa, J., Construction of a pair of practical *Nocardia-Escherichia coli* shuttle vectors. *Jpn. J. Infect. Dis.* **2007,** 60, (1), 45-47.
4. Jiao, S.; Chen, J.; Yu, H.; Shen, Z., Tuning and elucidation of the colony dimorphism in Rhodococcus ruber associated with cell flocculation in large scale fermentation. *Appl. Microbiol. Biotechnol.* **2017,** 101, (16), 6321-6332.
